# Structure and transcriptional regulation of the major intrinsic protein gene family in grapevine

**DOI:** 10.1186/s12864-018-4638-5

**Published:** 2018-04-11

**Authors:** Darren Chern Jan Wong, Li Zhang, Isabelle Merlin, Simone D. Castellarin, Gregory A. Gambetta

**Affiliations:** 1 Wine Research Centre, University of British Columbia, 2205 East Mall, Vancouver, BC V6T 0Z4 Canada; 2 Bordeaux Science Agro, Institut des Sciences de la Vigne et du Vin, Ecophysiologie et Génomique Fonctionnelle de la Vigne, UMR 1287, F– 33140 Villenave d’Ornon, France

**Keywords:** Aquaporin, Berry ripening, Cis-regulatory element, Promoter structure

## Abstract

**Background:**

The major intrinsic protein (MIP) family is a family of proteins, including aquaporins, which facilitate water and small molecule transport across plasma membranes. In plants, MIPs function in a huge variety of processes including water transport, growth, stress response, and fruit development. In this study, we characterize the structure and transcriptional regulation of the MIP family in grapevine, describing the putative genome duplication events leading to the family structure and characterizing the family’s tissue and developmental specific expression patterns across numerous preexisting microarray and RNAseq datasets. Gene co-expression network (GCN) analyses were carried out across these datasets and the promoters of each family member were analyzed for cis-regulatory element structure in order to provide insight into their transcriptional regulation.

**Results:**

A total of 29 *Vitis vinifera* MIP family members (excluding putative pseudogenes) were identified of which all but two were mapped onto *Vitis vinifera* chromosomes. In this study, segmental duplication events were identified for five plasma membrane intrinsic protein (PIP) and four tonoplast intrinsic protein (TIP) genes, contributing to the expansion of PIPs and TIPs in grapevine. Grapevine MIP family members have distinct tissue and developmental expression patterns and hierarchical clustering revealed two primary groups regardless of the datasets analyzed. Composite microarray and RNA-seq gene co-expression networks (GCNs) highlighted the relationships between MIP genes and functional categories involved in cell wall modification and transport, as well as with other MIPs revealing a strong co-regulation within the family itself. Some duplicated MIP family members have undergone sub-functionalization and exhibit distinct expression patterns and GCNs. Cis-regulatory element (CRE) analyses of the MIP promoters and their associated GCN members revealed enrichment for numerous CREs including AP2/ERFs and NACs.

**Conclusions:**

Combining phylogenetic analyses, gene expression profiling, gene co-expression network analyses, and cis-regulatory element enrichment, this study provides a comprehensive overview of the structure and transcriptional regulation of the grapevine MIP family. The study highlights the duplication and sub-functionalization of the family, its strong coordinated expression with genes involved in growth and transport, and the putative classes of TFs responsible for its regulation.

**Electronic supplementary material:**

The online version of this article (10.1186/s12864-018-4638-5) contains supplementary material, which is available to authorized users.

## Background

Aquaporins are channel-forming transmembrane proteins present in plasma and intracellular membranes in all eukaryotes and most prokaryotes [1]. Initially, aquaporins’ water transport capabilities were discovered and functionally characterized in human red blood cells [2–4] and later in plants (*Arabidopsis thaliana*) with the functional characterization of a vacuolar water-transporting protein, γ-TIP [5]. After the discovery of plant aquaporins, many studies have been conducted in order to elucidate their structure, function, and regulation across numerous plant species [6–8]. Aquaporins were first characterized as water channels, but they are also recognized to contribute to the transport of other small neutral molecules (e.g., glycerol, urea, boric acid, silicic acid), gases (e.g. CO_2_, ammonia), and even ions under certain circumstances [7–10].

Aquaporins fall within an ancient superfamily of membrane proteins called the major intrinsic proteins (MIPs). The MIP family consists of a large number of homologs, and can be subdivided into four major subfamilies based on sequence similarity, which may also indicate their sub-cellular localizations [11, 12]. The plasma membrane intrinsic proteins (PIPs), the tonoplast intrinsic proteins (TIPs), and the nodulin26-like intrinsic proteins (NIPs), comprise the major subfamilies [6, 8, 13]. These three groups of aquaporins have been intensively studied and well-documented. The small basic intrinsic proteins (SIPs) include only a few isoforms localized in the ER (e.g., 3 homologs in *Arabidopsis*) [9, 14]. In addition to these four well-conserved subfamilies present in all plant species, several additional novel types of aquaporins have been distinguished but with a less ubiquitous presence among plant species. For example, the uncategorized X intrinsic proteins (XIPs) were recently discovered but are absent in some higher plants, including Arabidopsis [15–17]. The GlpF-like intrinsic proteins and the hybrid intrinsic proteins were discovered in moss and algae, but are absent in vascular plants [9, 13].

Aquaporins facilitate water transport through plant cells and tissues and play critical rolls in numerous physiological processes. At the cell level, aquaporins act in osmoregulation, reactive oxygen species signaling, and intracellular transport and storage processes [9]. At the tissue and organ level, aquaporins contribute to plant water uptake in roots [18] and facilitate changes in leaf hydraulic conductance [19]. Additionally, aquaporins modulate changes in plant water relations in response to abiotic stress, including drought, salt, and temperature [9]. In fleshy fruit, there is evidence that aquaporins may contribute to ripening processes in tomato [20] and grape [21, 22].

The structure of the MIP gene family, like many plant gene families, has undergone numerous gene duplications resulting in groups of closely related isogenes [11, 23]. These closely related isogenes can have overlapping patterns of expression, or can have undergone sub-functionalization taking on specific developmental and/or tissue related expression patterns [24]. This is certainly the case for MIP family members where many isogenes display tissue and/or developmentally specific expression patterns. Tissue specific expression of MIP isogenes has been observed in numerous species including poplar [25], corn [1, 26, 27], rice [10, 28], Arabidopsis [29], and tomato [20] among other species. On an even finer scale specific isogenes have been associated with specific cell types within organs [19, 30], although most previous studies were not comprehensive across all MIP family members or across organs/tissues.

Grapevine is a plant species of economic and cultural importance and one of the first to have its genome sequenced [31]. This information allowed for the characterization of large gene families such as the MIP family, and indeed this genome information was immediately utilized to integrate cDNA and genome information in characterizing the MIP family members in grapevine [32]. Since then the original Pinot noir genome has been greatly improved and there has been a wealth of microarray and RNAseq studies examining a plethora of conditions (organ specificity, developmental stages, biotic and abiotic stresses, agronomical practices, etc.). Furthermore, new tools and approaches have been developed for analyzing the nature of genome duplications [33], as well as gene expression and cis-regulatory element structure [34]. These improvements allow for a more comprehensive analysis of the grapevine MIP gene family.

In the current study we utilized new tools and approaches to characterize the structure and transcriptional regulation of the MIP gene family in grapevine. We reassessed the MIP family members with the updated genome information describing the putative genome duplication events leading to the current family structure. The expression of family members was then assessed across numerous preexisting microarray and RNAseq datasets in order to determine their tissue and developmental specific expression patterns. Co-expression analyses were carried out across these datasets to determine relevant co-regulation patterns within the MIP gene family and within the transcriptome as a whole. Finally, the promoters of each family member were analyzed for cis-regulatory element structure in order to provide insight into the possible transcriptional regulation of each member.

## Methods

### Dendrogram construction and gene duplication classification

The grapevine MIP gene family sequences were retrieved from the ORCAE 12× grapevine annotation V2 (http://bioinformatics.psb.ugent.be/orcae/) through a combination of keyword and BLAST searches (using default parameters). For the truncated sequences the surrounding regions were visually inspected for sequence homology to ensure the predicted open reading frames were correct. Gene nomenclature was created following the guidelines established in Grimplet et al. 2014 [35]. Orthology assignment between predicted grapevine MIPs with Arabidopsis proteins was performed using the Conditional Reciprocal Best (CRB)-BLAST method using default settings [36].

Multiple sequence alignments and dendrogram constructions were carried out with Phylogeny.fr [37]. The family was split into sub-families for alignments in order to avoid artifacts caused by aligning large groups [38]. Sequences were aligned with MUSCLE (v3.8.31) using the highest accuracy default settings. After alignment gaps and/or poorly aligned regions were removed using Gblocks (v0.91b) using the following parameters: minimum length of a block after gap cleaning = 5, no gap positions were allowed in the final alignment, all segments with contiguous nonconserved positions bigger than 8 were rejected, minimum number of sequences for a flank position = 55%. Dendrograms were reconstructed using the maximum likelihood method implemented in the PhyML program (v3.1/3.0 aLRT) using default settings. Reliability for internal branch was assessed using the bootstrapping method (100 bootstrap replicates). Dendrograms were drawn with TreeDyn (v198.3).

Analysis of genome structure and duplication analysis was performed using MCScanX [33] using previously established parameters [39]. Information pertaining to the gene duplication type (i.e. singleton, dispersed, proximal, tandem, and segmental; for definition see http://chibba.pgml.uga.edu/mcscan2/), detected collinear pairs, and tandem/proximal gene duplicate groups were further analyzed. Briefly, all genes are initially assigned as ‘singletons’ and ranked (in ascending order) following their positions along chromosomes. Next, all-vs-all BLASTP is performed and results evaluated. The genes with BLASTP hits to other genes are assigned with ‘dispersed’ duplicates. Any two genes are assigned ‘proximal’ duplicates if the difference between gene ranks are < 20 while a rank = 1 between two genes are assigned as ‘tandem’ duplicates. Anchor genes within collinear blocks are assigned as ‘WGD/segmental’ duplicates. In the event where a gene have multiple BLASTP hits, assignment of duplication mode will be in the order of priority beginning with WGD/segmental followed by tandem, proximal, and finally dispersed duplication.

### RNA-seq data analysis

Publicly available grapevine next generation sequencing datasets were downloaded from NCBI Sequence Read Archive (http://www.ncbi.nlm.nih.gov/sra). Raw fastq reads (single- and paired-end) were extracted using SRA toolkit fastq-dump. Read trimming and quality filtering of reads (single- and paired-end) were performed with Trimmomatic v0.36 [40], with the following parameters; LEADING:20, TRAILING:20 SLIDINGWINDOW:4:20, MINLEN:40, AVGQUAL:20. Alignment of filtered reads towards the 12× grapevine reference genome [31] was performed using HISAT2 v2.0.5 [41] with default parameters. Gene-level count summarization was performed using featureCounts [42] using the grapevine 12× v1 (http://genomes.cribi.unipd.it/) reference annotation and subsequent transcript abundance, expressed as fragments per kilobase of transcript per million mapped reads (FPKM), estimated with edgeR [43].

### Gene co-expression network analysis

Two mutual rank (MR) [44] gene co-expression networks (GCN) were constructed, one based on RNA-seq data analyzed in this study and another based on the 29 K NimbleGen whole-genome microarray data. RNA-seq GCN was constructed using log-transformed FPKM values of 29, 970 genes × 237 conditions obtained in this study. Experiment accessions and publication references of analyzed data can be found in Additional file 1: Table S11. Microarray GCN was constructed from an updated input matrix of Wong et al. 2016 [39] containing 29, 000 genes × 358 conditions, an additional of 139 conditions compared to the previous study. Gene-centric co-expression clusters were created for each MIP gene from both RNA-seq and microarray GCNs by considering their top 100 co-expressed genes (ranked by MR value). Visualization of the various MIP networks was carried out in Cytoscape v3.0 [45]. Enrichment of MapMan BIN categories within co-expression clusters were evaluated for enrichment using Fisher’s exact test adjusted with false discovery rate (FDR) for multiple hypothesis correction according to Wong et al. 2016 and 2017 [34, 39]. MapMan BIN categories were considered significantly enriched within co-expression clusters with a FDR < 0.05.

### Cis-regulatory element analysis in promoter region

The frequencies and position information of selected cis-regulatory elements (CREs) within 1 kb promoter region from the transcription start site of MIP genes were obtained from Wong et al. 2017 [34] and further analyzed for position bias *Z-score* considering MIP gene family as a whole/only [46]. The *Z-score* for each CRE was determined using the equation: Z-score = (*L*/2 + *p*)/ √[((*L*- *l* + 1)^2–1)/n]. This strategy takes into account the length of the promoter, *L*; length of the CRE, *l*; total number of CRE hits present in all promoters, *n*; and mean position from all identified CRE hits, *p*. Consideration of these well-established criteria as a whole improves the likelihood of identifying bona fide CREs in selected promoter groups [46].

## Results

### Family structure

**Fig. 1:**
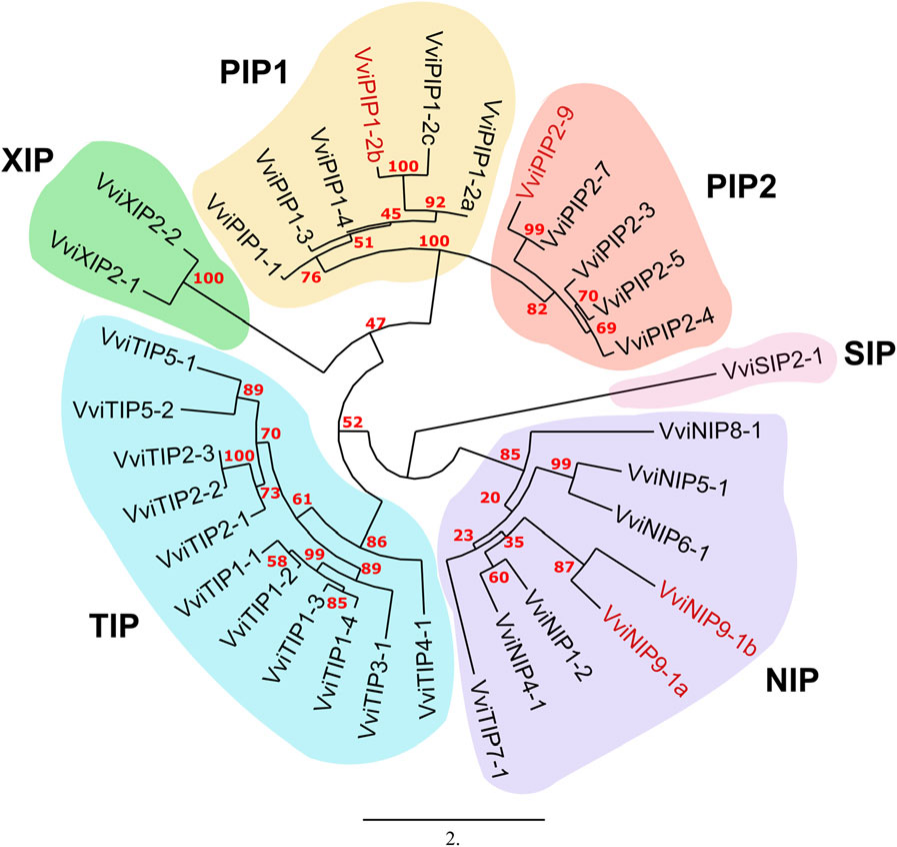
Protein sequence relationships within the *Vitis vinifera* MIP family. The six major MIP sub-families are shown: PIP1s, PIP2s, TIPs, NIPs, SIPs, and XIPs. Red numbers represent bootstrap values (100 bootstrap replicates). Putative pseudogenes are shown in red. Detailed accession, homology, and duplication information is presented in Additional file 1: Table S1

**Fig. 2:**
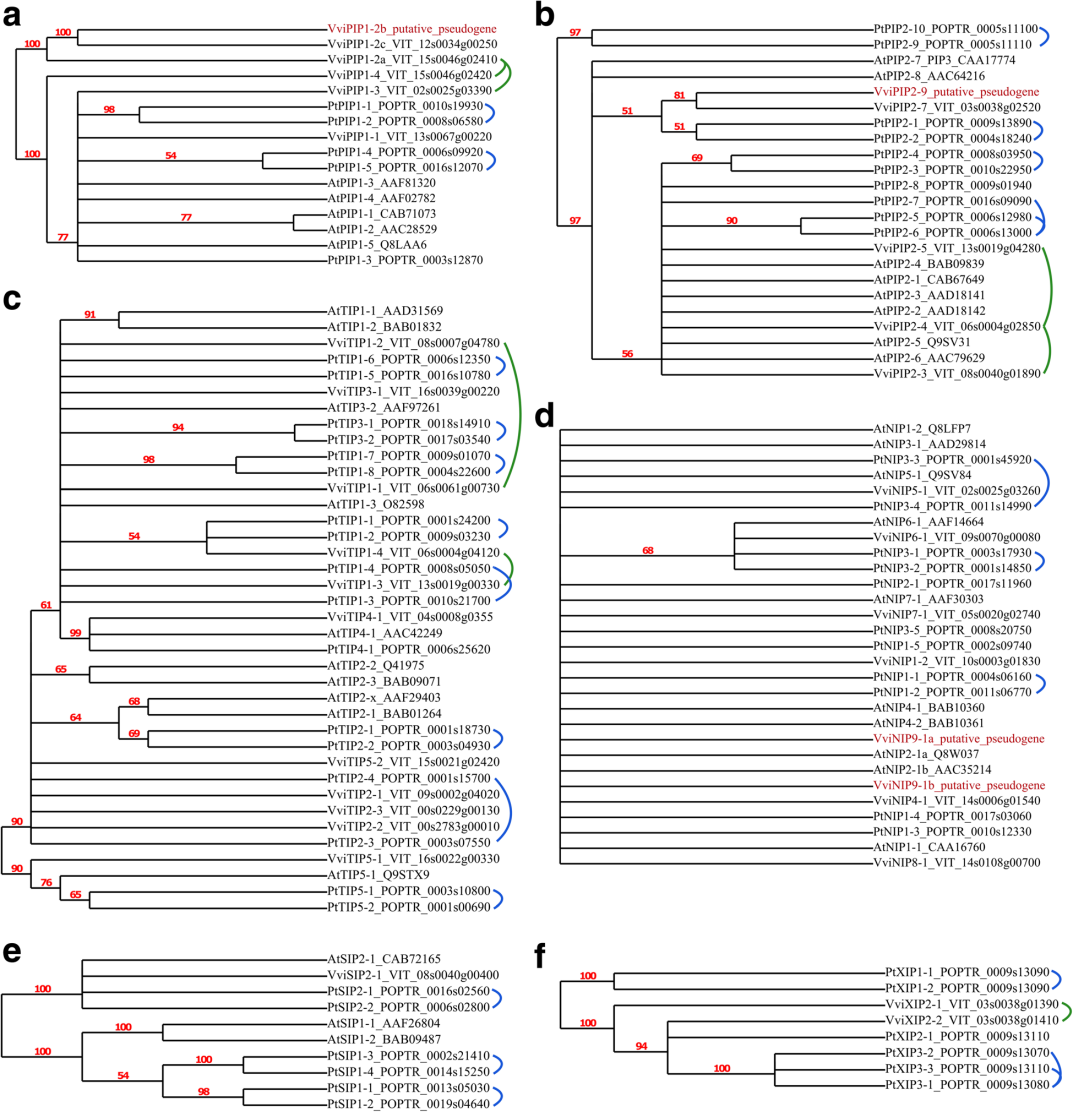
Protein sequence relationships between the *Vitis vinifera*, Arabidopsis, and poplar MIP families. Six major MIP sub-families: PIP1s (**a**), PIP2s (**b**), TIPs (**c**), NIPs (**d**), SIPs (**e**), and XIPs (**f**). Red numbers represent bootstrap values and the tree was collapsed for all bootstrap values under 50 (100 bootstrap replicates). Linked proteins represent gene duplications for *Vitis vinifera* (green links) and poplar (blue links as detailed in [25]). Putative pseudogenes are shown in red. Detailed accession, homology, and duplication information is presented in Additional file 1: Table S1

A total of 33 *Vitis vinifera* MIP family members were identified (Fig. 1; Additional file 1: Table S1). Of these 33 family members we designated 4 of them (VviPIP1–2b, VviPIP2–9, VviNIP9-1a and b) as putative pseudogenes (shown in red in Figs. 1 and 2) because they were both truncated and not expressed in any of the RNAseq datasets we analyzed. These 4 genes were excluded from subsequent analyses in this work. Direct orthologous relationships between *Vitis vinifera*, poplar, and Arabidopsis are extremely difficult to establish as evidenced by the numerous collapsed dendrogram branches (Fig. 2). We performed additional reciprocal BLAST analyses between the Arabidopsis and *Vitis vinifera* genes to aid in orthology identification, but again in many cases the orthology could not be resolved (Additional file 1: Table S1; column J “ambiguous”).

We examined the nature of duplication events contributing to the size of the grapevine MIP gene family (Fig. 2; Additional file 1: Table S1). A total of 27 of 29 grapevine MIP genes were successfully mapped on all 19 grapevine chromosomes. Location of the remaining two, *VviTIP2–2* and *VviTIP2–3*, remains unresolved based on the current 12× genome assembly. In this study, segmental (9 of 29) duplication events were identified for five PIP (*VviPIP1–2a*, *VviPIP1–3*, *VviPIP2–3*, *VviPIP2–4*, *VviPIP2–5*) and four TIP (*VviTIP1–1*, *VviTIP1–2*, *VviTIP1–3*, *VviTIP1–4*) genes, contributing to the expansion of PIPs and TIPs in grapevine. For example, *VviPIP2–4* is collinear to both *VviPIP2–3* and *VviPIP2–5* and *VviTIP1–1* is collinear with *VviTIP1–2*. The PIP duplicates are located on collinear blocks on chromosomes 2/15 and 6/8/13 while TIP duplicates are on chromosome 6/8/13. Meanwhile, tandem duplication was observed for *VviPIP1–4* and *VviPIP1–2a*, where the latter is also a segmental duplicate with *VviPIP1–3*. Proximal duplication was observed for *VviXIP2–1* and *VviXIP2–2* where the two are separated by a disease resistance protein. The remaining were classified as dispersed (16 of 29) duplicates whereby the specific mode of duplication is unclear (i.e. other than segmental, tandem, and proximal duplication) and no MIPs were identified as singletons.

### Tissue and developmental specific expression and sub-functionalization

**Fig. 3:**
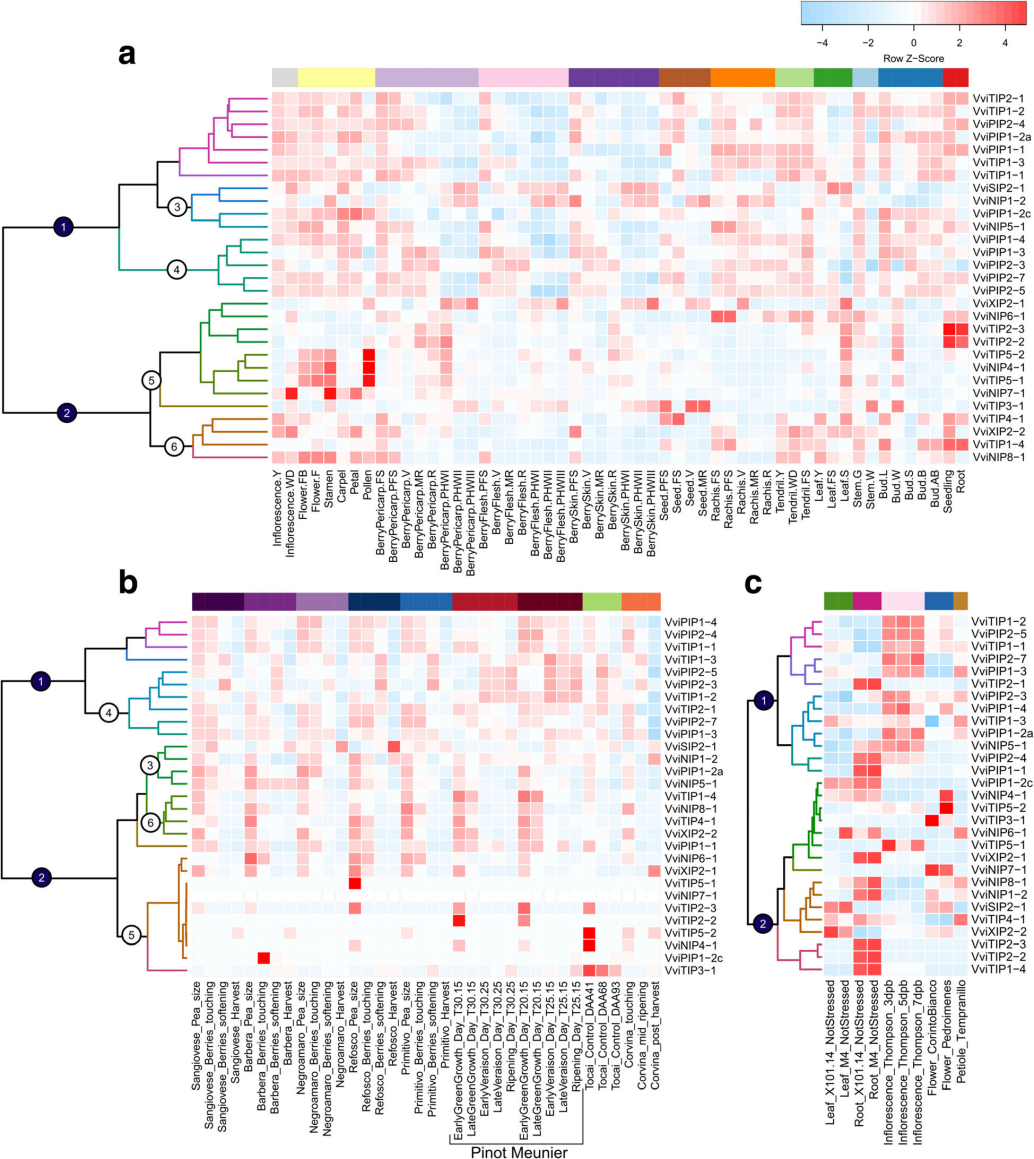
Expression of the grapevine MIP gene family across the NimbleGen grapevine expression atlas (**a**) and various other RNAseq datasets in berries (**b**) and other organs (**c**; note only “control” states are compared). Colored bars group like tissues or genotypes. Heatmap represents the Z-score according to the scale depicted. Like groupings are numbered for clarity (Groups 1& 2, numbered blue circles, Sub-groups 3–6, numbered white circles). RNAseq experiment accessions and publication references of analyzed data can be found in Additional file 1: Table S11. Raw Z-score values can be found in Additional file 1: File S2 and S3

Tissue and developmental specific expression profiles of the MIP family members were assessed by examining their expression profiles across the nimblegen grapevine expression atlas [47] (Fig. 3a; Additional file 1: Table S2) and a wide range of existing RNA-seq datasets (Fig. 3b and c; Additional file 1: Table S3). Grapevine MIP family members have distinct tissue and developmental expression patterns. Hierarchical clustering revealed two primary groups (Fig. 3 groups 1 and 2) that were similar regardless of the datasets analyzed. Comparing just the expression atlas (Fig. 3a) with grape berry RNAseq datasets (Fig. 3b), the composition of several subgroups are nearly identical (Fig. 3 sub-groups 3–6).

Generally speaking MIP family members are ubiquitously expressed across tissues, although their expression differs across developmental stages (Fig. 3a). This is true within subfamilies as well with particular isogenes being expressed in almost all tissues, again at specific developmental stages. The inflorescence and flower parts tend to have high levels of MIP expression across the whole family. The primary groups described above (Fig. 3a groups 1 and 2) generally differ in that group 1 is more highly expressed.

Expression across berry development was examined more closely because of the lack of information on aquaporins’ role in fruit development as well as the wealth of datasets available. Of the two primary groups (Fig. 3b groups 1 and 2), group 1 has a much more dynamic expression pattern across berry development regardless of tissue or genotype. In most cases these family members are highly expressed early in berry development and down-regulated as development progresses. However, several members of sub-groups 3 and 4 are up-regulated at the onset of ripening and later during maturation of the berry (Fig. 3b; e.g. *VviPIP2–3, VviPIP2–5, VviTIP1–2, VviTIP1–3*). In contrast to group 1, group 2 is less dynamic across berry development with a few exceptions, most notably a cluster of family members that exhibit pericarp-specific expression (Fig. 3a sub-group 5).

Duplicated MIP family members exhibit sub-functionalization, at least at the level of their transcriptional regulation. For example, *VviXIP2–1* and *VviXIP2–2* have distinct expression patterns across a variety of datasets (Fig. 3). This is also true for other examples such as for *VviPIP1–4* and *VviPIP1–2a*. However some duplicated family members exhibit less distinct expression patterns such as *VviTIP1–1* and *VviTIP1–2*.

### Enriched functional categories in grapevine MIP gene co-expression networks

To infer the most representative biological functions of this mid-sized gene family, we queried two condition-independent gene co-expression network (GCNs) using individual MIP genes as ‘guides’ separately (Additional file 1: Table S4 and Additional file 1: Table S5) and analyzed their top 100 correlators in detail for biological pathway enrichment (Additional file 1: Table 6 and Additional file 1: Table S7).

**Fig. 4:**
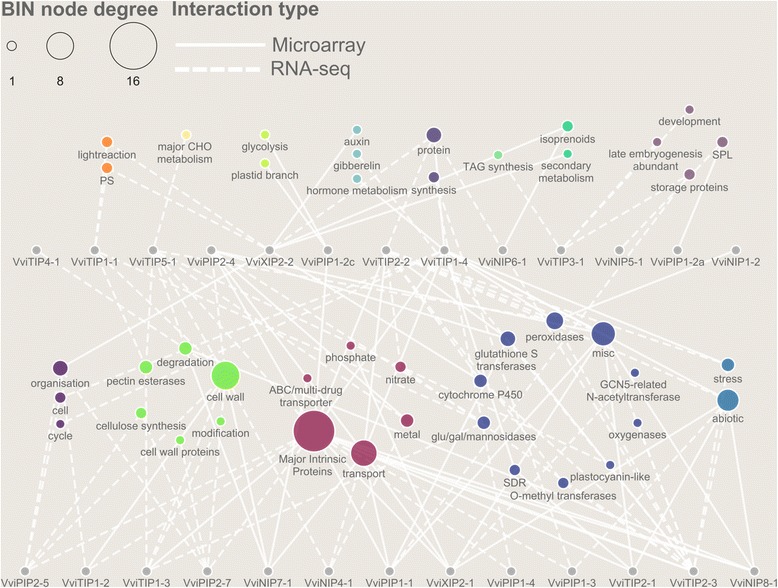
Enriched functional categories identified from composite microarray and RNA-seq MIP gene co-expression networks. Node color represents the parent functional BIN category at BIN depth ≤ 1. Circle size represents the frequency of microarray and/or RNA-seq MIP gene co-expression networks enriched with the corresponding functional BIN category. Solid and dashed edges represent enriched functional BIN category in relevant microarray and RNA-seq MIP gene co-expression networks, respectively

In a composite microarray and RNA-seq GCN highlighting MIP genes and their high-level (BIN depth ≤ 1) enriched functional categories (Fig. 4), BIN categories such as transport (BIN34), cell wall (BIN10), miscellaneous enzyme reactions (BIN26) were commonly enriched in MIP co-expression networks. Conversely, categories such as stress (BIN20), cell organization (BIN31), protein metabolism (BIN29), and development (BIN33) were only enriched in specific MIP networks. Specific categories within transport, especially Major Intrinsic Proteins (BIN34.19), were enriched in 17 MIP co-expression networks. Other categories within transport such as ABC/multi-drug transporter (BIN34.16), phosphate (BIN34.7), nitrate (BIN34.4), metal (BIN34.12) were enriched in one (*VviNIP7–1*), one (*VviXIP2–1*), two (*VviTIP2–3*, *VviTIP2–2*), and three (*VviPIP2–4*, *VviTIP5–1*, *VviNIP4–1*) MIP GCNs, respectively (Additional file 1: Table S6 and Additional file 1: Table S7).

Enrichment of cell wall (BIN10) categories observed in many MIP co-expression networks was unexpected (Fig. 4, Additional file 1: Table S6 and Additional file 1: Table S7). In particular, genes encoding cell wall proteins (BIN10.5), degrading enzymes (BIN10.6), pectin esterases (BIN10.8), cellulose synthesis (BIN10.2), and modification (BIN10.7) belong to categories that were enriched in three (*VviTIP5–2*, *VviPIP2–7*, *VviTIP1–1*), four (*VviNIP7–1*, *VviNIP8–1*, *VviTIP5–1*, *VviTIP1–3*), three (*VviTIP5–1*, *VviTIP1–1*, *VviTIP1–3*), two (*VviPIP2–7*, *VviPIP2–5*), and one (*VviTIP1–3*) MIP GCNs, respectively. The two categories that deserve attention within the miscellaneous (BIN26) category relate to the enrichment of glutathione-S-transferase and peroxidase co-expressed genes in four (*VviTIP2–3*, *VviTIP2–2*, *VviPIP2–4*, *VviPIP1–1*) and five (*VviTIP1–4*, *VviNIP1–2*, *VviTIP2–3*, *VviTIP2–2*, *VviTIP5–1*) MIP GCNs, respectively. Meanwhile, seven MIP GCNs (*VviNIP8–1*, *VviXIP2–1*, *VviTIP1–4*, *VviTIP2–1*, *VviPIP1–3*, *VviTIP2–2*, *VviTIP2–3*) enriched with abiotic stress related genes (BIN20.2) are also of interest.

### Divergence of enriched functional categories in gene co-expression networks of grapevine MIP duplicates

As a significant proportion of grapevine MIP duplicates showed sub-functionalization of gene expression in a tissue-specific manner (Fig. 3), we compared the enriched functional categories in the GCNs (Additional file 1: Table S6 and Additional file 1: Table S7) of MIP duplicate pairs (Additional file 1: Table S1). GCNs of duplicates such as *VviXIP2–1* and *VviXIP2–2* have totally distinct enriched categories. While major intrinsic proteins (BIN34.19) and abiotic stress (BIN20.2) genes were enriched in *VviXIP2–1* GCNs, the latter two categories were absent in *VviXIP2–2* GCN. Instead, genes involved in the light reaction of photosynthesis (BIN1.1), isoprenoid metabolism (BIN16.1), auxin metabolism (BIN17.2), and CYP450-coding genes (BIN26.1) were enriched in *VviXIP2–2* GCNs. In another example, GCNs of *VviTIP1–3* and *VviTIP1–4* duplicate pairs have in common enrichment for major intrinsic proteins (BIN34.19), however, enrichment of cell wall pectin esterases (BIN10.8) and modification (BIN10.7) was observed for *VviTIP1–3* while abiotic stress (BIN20.2), cell cycle (BIN31.3), and hormone (i.e. JA and ABA) metabolism (BIN17) functional categories were among the many categories enriched in the GCN of its duplicate *VviTIP1–4*.

Conversely, duplicated family members that exhibit less divergent expression profiles such as *VviPIP2–4* and *VviPIP2–5* showed more commonalities. Both *VviPIP2–4* and *VviPIP2–5* have a common enrichment for cell wall (BIN10) and major intrinsic proteins (BIN34.19), albeit some differences were apparent such as enrichment for glutathione S transferases (BIN26.9) and cell organization (BIN31.1) in *VviPIP2–4* and *VviPIP2–5*, respectively (Additional file 1: Table S6 and Additional file 1: Table S7). Similarly, *VviTIP1–1* and *VviTIP1–2* share enrichment for cell wall (BIN10) related genes, but categories related to light reaction (BIN1.1) and cell organization (BIN31.1) were enriched in *VviTIP1–1* and *VviTIP1–2*, respectively.

### Cis-regulatory element structure of grapevine MIP promoters

**Fig. 5:**
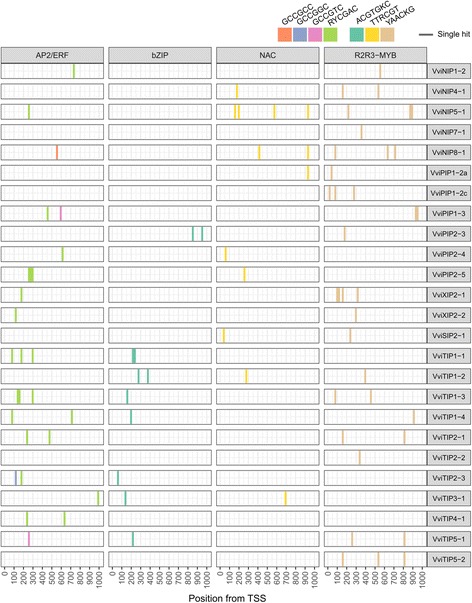
Number and location of highlighted cis-regulatory elements in the promoters of grapevine MIP family members. The AP2/ERF (orange, blue, pink, green), bZIP (turquoise), NAC (yellow), and R2R3–MYB (beige) were amongst top 10 (of 222) most highly ranked CREs (6- to 8-mer in length) based Z-score. Each occurrence of the CRE is noted at its position with the appropriate colored line. Complete promoter CRE data can be found in Additional file 1: Table 8 and Additional file 1: Table S9

Genome-wide analysis in grapevine promoters have highlighted many CREs possessing strong position bias towards the transcription start site (TSS) which were implicated in a variety of grapevine development and stress responses [34]. To determine which CREs are biologically relevant for the regulation of grapevine MIPs we extracted the distribution patterns of 222 CREs (6- to 8-mer in length) in the promoter region for grapevine MIPs (Additional file 1: Table S8) selected from Wong et al. 2017 [34]. The frequency of occurrence, the median position of occurrence, and position bias Z-score were evaluated. On these subset of MIP genes, 6-mer and 7-mer CREs namely RYCGAC, YAACKG, TTRCGT, and ACGTGKC were amongst top 10 most highly ranked CREs based Z-score (Fig. 5**;** Additional file 1: Table S9). The most highly ranked CRE, the RYCGAC – part/variant of the dehydration-responsive element (DRE)/C-repeat elements/low-temperature-responsive element [48] – were present in 14 MIP promoters (∑ _hits_: 23, M _position_: 262) followed by YAACKG CRE – part/variant of the type I R2R3-MYB recognition sites [49] – that were present in 19 MIP promoters (∑ _hits_: 35, M _position_: 315). The TTRCGT CRE – the major NAC TF recognition sites [50] – was also ranked highly and was present in 9 MIP promoters (∑ _hits_: 13, M _position_: 286). Longer CREs such as ACGTGKC [51] – a well-known ABA-responsive element (ABRE) – were present in 8 MIP promoter (∑ _hits_: 12, M _position_: 226). For most of these CREs, a position bias towards the TSS (M _position_ < 300) was also observed considering MIP genes.

### Enrichment of known cis-regulatory elements in grapevine MIP gene co-expression networks

**Fig. 6:**
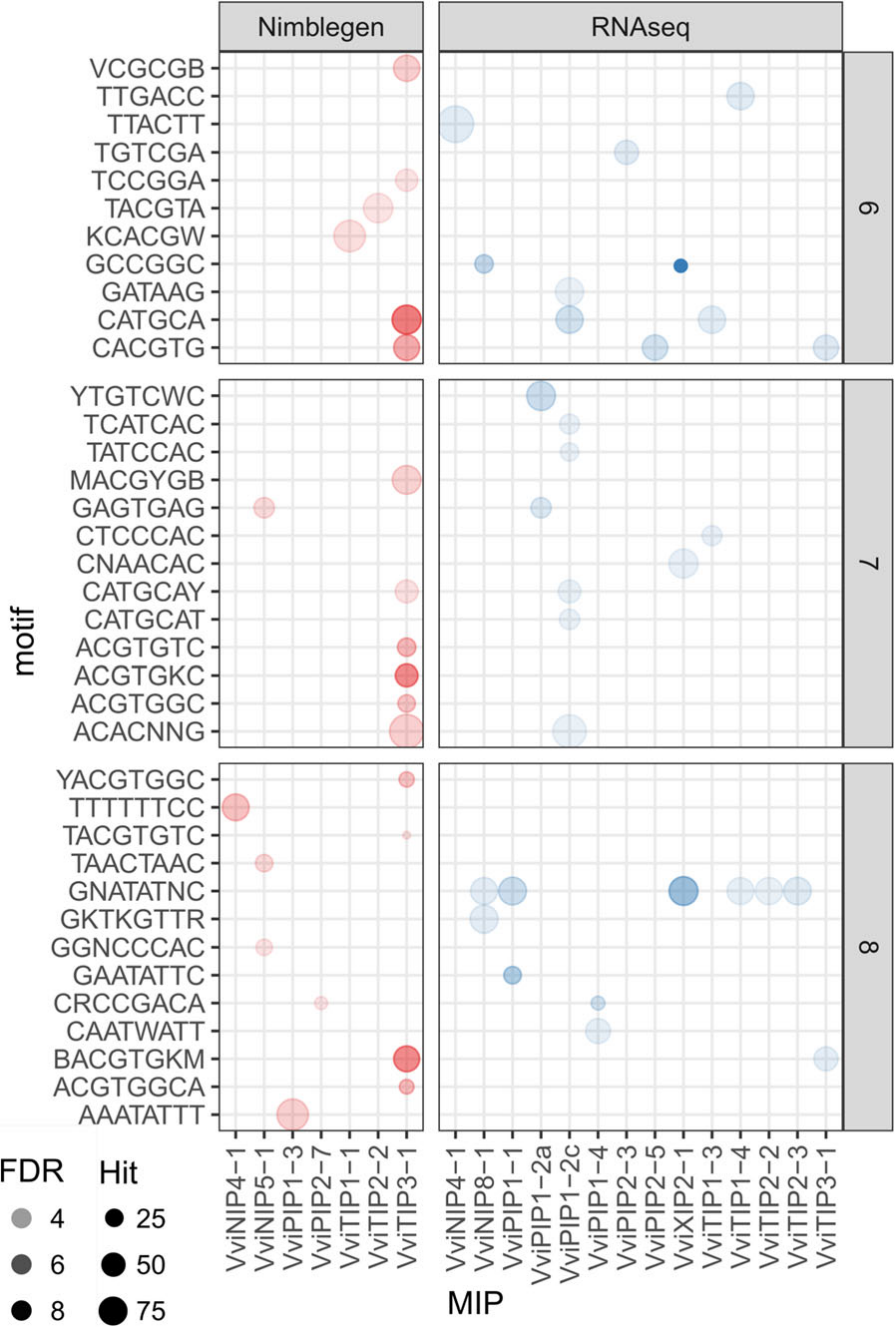
Enriched cis-regulatory elements within shared MIP co-expression networks. Enriched CREs between 6- and 8-mer are depicted as separate panels. Circle opacity represents the enrichment score (−log10 FDR values) of the corresponding enriched CRE and circle size of represents the total number of genes containing the enriched CRE

In this study, promoters of genes within MIP GCNs were also tested for enrichment for known CREs in order to identify putative shared TF families within the MIP GCNs that may be responsible for their transcriptional regulation. Nineteen MIP GCNs displayed significant enrichment (FDR < 0.01) for at least one CRE tested (Fig. 6; Additional file 1: Table S10). Of these, six MIP GCNs (i.e. *VviNIP8–1*, *VviPIP1–1*, *VviXIP2–1*, *VviTIP1–4*, *VviTIP2–2*, and *VviTIP2–3*) were commonly enriched for the PHR1-binding sequence (P1BS, GNATATNC). Several of these MIP GCNs were also co-enriched with other CREs. For example, AP2/ERF (GCCGGC) and R2R3-MYB (GKTKGTTR) related CREs were observed in the *VviNIP8–1* GCN along with related genes such as AP2/ERF TFs, *VviTOE3* (VIT_01s0026g01690), *VviERF1L4* (VIT_07s0005g03270), and *VviMYB82C* (VIT_11s0016g05690). Promoters of the *VviXIP2–1* GCN are also enriched for the GCCGGC CRE correlating with the presence of two AP2/ERF TFs, *VviTOE2* (VIT_14s0108g00050) and *VviTOE3* (VIT_01s0026g01690). Other co-enriched CREs of interest include HB (CAATWATT) and extended DRE elements (DEAR4, CRCCGACA) in promoters of the *VviPIP1–4* GCN, coincident with three HB TFs (VIT_08s0007g01290, VIT_16s0100g00670, VIT_18s0001g08410) and two AP2/ERF TFs *VviERF061* (VIT_02s0025g01360) and *VviERF022* (VIT_18s0001g05850).

Interestingly, *VviTIP3–1* and *VviPIP1 − 2c* were the two GCNs enriched with many of the CREs tested. For example, many ACGT-related (e.g. ACGTABREMOTIFA2OSEM, GADOWNAT, CACGTGMOTIF, ABREATCONSENSUS) and RY-related (i.e. RYREPEATBNNAPA, RYREPEATLEGUMINBOX) CREs, but also others involved in Ca2+/calmodulin signaling (CGCGBOXAT) and lateral organ boundary TF binding (TCCGGA) were enriched in the *VviTIP3–1* GCN specifically. A suite of TFs whose homologs target the latter CREs were also present reaffirming the biological relevance of this broad enrichment pattern (Fig. 6; Additional file 1: Table S10). This includes two B3 TFs, *VviABI3* (VIT_07s0005g05400) and *VviFUS3* (VIT_14s0068g01290) whose homologs/orthologs in Arabidopsis target the RY motif [52] and *VviABI5*/*VvbZIP25* (VIT_08s0007g03420) that targets various ACGT-related CREs.

## Discussion

### Expansion and sub-functionalization of the grapevine MIP family

The number of grapevine MIPs identified (33) is similar to the number identified in earlier versions of the grape genome assembly (29 MIPs, [32]), Arabidopsis (35 MIPs, [11]), and rice (33 MIPs, [10]). The MIP family is highly conserved and although many orthologous grapevine-Arabidopsis pairs were identified more than half of the orthologous relationships were impossible to resolve. The annotation and gene names presented here differ at times (8 of 33) from those established in Shelden et al. (2009) [32]. This is primarily due to a much improved genome assembly which allowed for the identification of previously unidentified family members, and improved computational methods for identifying the most likely Arabidopsis orthologs [36].

The grapevine MIP gene family has undergone a number of duplication events consistent with the highly duplicated nature of plant genomes and grapevine specifically [31]. The duplication events concerning segmental and tandem duplications identified in this study have also been reported for Arabidopsis [23] and rice [53, 54]. Nonetheless, novel duplication events involving *VviXIP2–1* and *VviXIP2–2* may be grapevine-specific.

It is commonplace that duplicated genes often take on different expression patterns, with respect to specific portions of development and/or location [24]. In the current study some duplicated MIP gene family members have distinct patterns of expression. It is likely that these duplicates have a similar protein function yet function in different contexts, for example VviXIP2–2 in leaves and VviXIP2–1 in roots (see Fig. 3c). In grapevine, several other gene families have a similar history of duplication and sub-functionalization [39, 55, 56]. Concerning fruit specifically, the expression of most MIP family members decreases as berry development progresses consistent with earlier studies [21, 22]. Grape berries become increasingly hydraulically buffered from the parent plant during ripening and this buffering is thought to result in part from decreases in hydraulic conductivity [21, 57]. This general downregulation of MIP family members during ripening may contribute to these decreases in berry hydraulic conductivity. In contrast, some specific isogenes (e.g. *VviPIP2–3* and *VviPIP2–5*; note *PIP2–5* was previously referred to as *PIP2–1*) show significant expression and even up-regulation throughout the later stages of berry development [21, 22]. Their role in fruit ripening remains unknown, but some have speculated that they may facilitate small ion transport and/or osmoregulation [58]. Fleshy fruits like grape berries undergo rapid growth and sugar accumulation during ripening and the role of aquaporins in mediating grape berry water relations is certainly worthy of further study [59].

The grapevine expression atlas [47] is a powerful dataset for examining tissue and developmental specific expression patterns however caution is warranted especially when examining highly conserved gene families. Microarray based expression analyses can be biased via cross-hybridization [60], and this is why it is important to include RNAseq based analyses as well. The results of the MIP family members presented here show strong parallels between both approaches suggesting that any potential cross-hybridization did not lead to erroneous results in the case of the expression atlas.

### Grapevine MIP co-regulation networks

Based on the ‘guilt-by-association’ principle, genes involved in related processes often share parallel expression dynamics across a wide range conditions including different organ/cell types, developmental stages, stress, and hormonal perturbations [61]. Gene co-expression networks (GCNs) analyses, which are built upon the ‘guilt-by-association’ principle, have been particularly useful for ascribing the most representative biological functions to both individual gene(s) [62–65] and large gene families [39, 66] in grapevine. This study highlights the strong co-expression relationships within the MIP family itself, and between MIP family members and genes involved various processes such as growth, cell-division, and cell redox homeostasis.

One of the strongest GCN relationships revealed in this study was that between the MIP family and genes involved in growth and transport processes, namely cell wall modification and cell expansion. Aquaporins have been implicated in the growth of rose flower petals and are part of a GCN associated with petal cell expansion [67]. In grape berries, targeted analyses of a limited number of aquaporins and cell wall metabolic genes were shown to have similar patterns of expression that correlated with growth [68]. The treatment of grape berries with exogenous ethylene stimulated growth and associated micro-array analyses revealed coordinated changes in the expression of both aquaporin and cell wall metabolic genes [69]. Among the cell wall metabolic genes identified by Schlosser et al. (2008) [68] and Chervin et al. (2008) [69] were the pectin esterases (BIN10.8) and cellulose synthesis (BIN10.2) identified in this study. The congruence between these previous studies and the more global a priori approach utilized here provides robust evidence for a functional link between these groups of genes.

Our GCN analyses also revealed a strong link between the MIP family and cell division (cell cycle, BIN31.3, and cell organization, BIN31.1). This is a relationship that has not been studied in plants apart from a few studies. Over-expression of tobacco NtTIP1;1 in cell culture enhanced cellular expansion and cell-division [70] and specific aquaporin isoforms have been associated with rapidly proliferating tissues in roots [71, 72]. Cell proliferation and growth involves the regulation of source-sink relationships intersecting with turgor driven growth, and one could speculate an important role for MIP family members in both of these processes. Outside of plants there is a growing body of work linking aquaporin function with the regulation of cell proliferation [73].

Another interesting GCN highlighted in this study was between the MIP family and cell redox homeostasis. The most obvious link between MIPs and redox homeostasis is the fact that many MIP isoforms transport hydrogen peroxide [74]. Therefore perhaps it should not come as a surprise that MIP family members would be among coordinated redox homeostasis genes. Links between aquaporin function and redox homeostasis are involved in the regulation of root water uptake under stress [75–77] but not necessarily through a transcriptional mechanism [78] and the same is true for pathogen responses [79]. Perhaps one of the most interesting observations is the nexus between cell expansion, cell division, and redox homeostasis [80], where aquaporins may play a cornerstone role in coordinating water fluxes and redox homeostasis in the control of growth.

### The diversity of bona fide cis-regulatory elements in grapevine MIP promoters

Regulation of plant MIP genes is still poorly understood. This study represents a first attempt of characterizing the CRE structure of the grapevine MIP family and identifying putative TFs responsible for its regulation. As limitations exists even for well-established statistical measures used for prioritizing CREs, combining several metrics may overcome potential caveats of each approach [34].

Recent studies have shown that the DRE and GCC-box (GCCGCC) core sequences are critical for the regulation of MIP genes by members of AP2/ERF subgroups I, IV, and V in several plants [81–84]. This is consistent with the highly prioritized DRE in MIP promoters among all other CREs (Fig. 5) and the co-regulation with AP2/ERF TFs including several predicted grapevine subgroup I, IV, and V members. Some of these regulatory relationships are conserved while many others are novel. Known relationships include co-regulation of a closely related grapevine homolog of Arabidopsis RAP2.11 (VIT_02s0025g03170) with *VviTIP2–1*, and co-regulation of *VviTIP3–1* with grapevine homolog of Arabidopsis DREB2D. These examples of co-regulation are consistent with known and predicted targets of Arabidopsis RAP2.11 [85] and DREB2D [86]. The DRE sites within *VviTIP2–1* and *VviTIP3–1* promoter may be important for its regulation in grapevine. Unexpectedly, GCC-box elements and other GCC-related CREs (GCCGGC, GCCGTC) were not found within most MIP promoters within 1000 bases from the TSS (Additional file 1: Table S8 and Additional file 1: Table S9). This observation might indicate potential divergence in AP2/ERF transcriptional regulatory networks involving MIP genes between plant species and the DRE may be preferred in grapevines, and/or that GCC-box and related CREs are located beyond the promoter regions analyzed in this study.

Several bZIP, NAC, and R2R3-MYB transcription factors have been shown to regulate specific MIP genes [67, 87, 88] consistent with many highly co-regulated TFs of these families in MIP subnetworks. Differences in the distribution of CREs present in MIP promoters were also observed (Fig. 5). PIP and TIP promoters contain mostly AP2/ERF and bZIP-related CREs while NIP promoters contain mostly NAC and R2R3-MYB-related CREs, suggesting some degree of transcriptional regulation specificity in grapevine aquaporin regulation.

Promoter analysis suggests that hormone metabolic pathways such as ABA and ethylene play an important role in the regulation of MIP genes. There is evidence for ethylene-regulated aquaporin expression in rose petals [67, 89] and aquaporin genes are among those regulated by exogenous ethylene treatment in grape berries [69]. Several studies demonstrate that ABA regulates the expression of numerous MIP family members [90–92]. However, it is important to point out that short-term modulation of aquaporin activity via ABA, and possibly other hormones such as ethylene, likely occurs at the post-translational level [93, 94]. In grapevine, ABA has been shown to differentially regulate the same aquaporin isogene (*VviTIP1–1*, VIT_06s0061g00730) depending on the organ [95]. These complex relationships involved in the hormonal regulation of aquaporin gene expression require further study.

The promoters of genes in six MIP GCNs were also commonly enriched for the PHR1-binding sequence (P1BS, GNATATNC). The cognate sequence, such as GAATATTC, is known to be bound by members of the MYB (GARP, G2) TF and is related to the regulation of transcriptional repressors [96] and accordingly no homologs of MYB (GARP, G2) TFs were represented in respective MIP GCNs. The enrichment of this CRE may suggest a potential role of large-scale transcriptional repression in the regulation of MIPs. Conversely, for many other CREs enrichment profiles were often accompanied by the presence of TF families known to target them (Fig. 6, Additional file 1: Table S10) suggesting a role of transcriptional activation of MIP and co-regulated genes. The diversity of enriched CREs also highlights that in addition to the those shown to be directly implicated in MIP regulation such as AP2/ERF, bZIP, NAC, and R2R3-MYB TFs, regulation of MIPs may involve more TF families than previously described. Several genes that belong to HB, LBD, and B3 TF families may also represent novel candidate regulators of grapevine MIP and co-regulated genes.

## Conclusions

The current work utilized the most high quality and up-to-date genome information in characterizing the grapevine MIP gene family, its structure, and the putative duplication events involved in its evolution. When paired with the GCN analyses conducted here we identified those MIP family members that have undergone duplication and sub-functionalization through characterizing the tissue and developmental specific expression patterns across the family. GCN analyses revealed several interesting relationships between MIP family members and genes involved in cell expansion, cell division, and transport processes. Characterizing the cis-regulatory elements in grapevine MIP promoters along with associated GCN members identified the putative classes of TFs responsible for the regulation of the family and their associated GCNs. Combining phylogenetic analyses, gene expression profiling, GCN analyses, and CRE enrichment, this study provides a comprehensive overview of the structure and transcriptional regulation of the grapevine MIP family. These results can help guide future studies aimed at understanding the role of specific transcription factors in controlling the diverse expression patterns within the MIP family.

## Additional file

Additional file 1: Supplementary Tables. (XLSX 748 kb)

## Abbreviations

CRE: Cis-Regulatory Element
DRE: Dehydration-Responsive Element
GCN: Gene Co-expression Network
MIP: Major Intrinsic Protein
NIP: Nodulin26-like Intrinsic Proteins
PIP: Plasma membrane Intrinsic Protein
SIP: Small basic Intrinsic Proteins
TIP: Tonoplast Intrinsic Protein
TSS: Transcription Start Site
XIP: X Intrinsic Proteins
